# Alterations in microRNA of extracellular vesicles associated with major depression, attention-deficit/hyperactivity and anxiety disorders in adolescents

**DOI:** 10.1038/s41398-023-02326-4

**Published:** 2023-02-06

**Authors:** Jessica Honorato-Mauer, Gabriela Xavier, Vanessa Kiyomi Ota, Samar Nasser Chehimi, Fernanda Mafra, Cássia Cuóco, Lucas Toshio Ito, Rafaella Ormond, Paula Fontes Asprino, Adrielle Oliveira, Amanda Victoria Gomes Bugiga, Ana Claudia Torrecilhas, Rodrigo Bressan, Gisele Gus Manfro, Euripedes Constantino Miguel, Luis Augusto Rohde, Pedro Mario Pan, Giovanni Abrahão Salum, Renata Pellegrino, Sintia Belangero, Marcos Leite Santoro

**Affiliations:** 1 Disciplina de Genética, Departamento de Morfologia e Genética - Universidade Federal de São Paulo (UNIFESP), São Paulo, Brazil; 2 Laboratório de Neurociências Integrativas (LiNC - UNIFESP), São Paulo, Brazil; 3 Center for Applied Genomics, The Children’s Hospital of Philadelphia (CHOP), Philadelphia, PA USA; 4 Hospital Sírio-Libanês, São Paulo, Brazil; 5 Departamento de Psiquiatria, Universidade Federal de São Paulo (UNIFESP), São Paulo, Brazil; 6 Laboratório de Imunologia Celular e Bioquímica de Fungos e Protozoários, Departamento de Ciências Farmacêuticas, Universidade Federal de São Paulo (UNIFESP), Diadema, Brazil; 7 Departamento de Psiquiatria, Universidade Federal do Rio Grande do Sul, Hospital de Clínicas de Porto Alegre, Porto Alegre, Brazil; 8 National Institute of Developmental Psychiatry, CNPq, São Paulo, Brazil; 9 Departamento de Psiquiatria, Faculdade de Medicina da USP- FMUSP, Instituto de Psiquiatria do HCFMUSP, São Paulo, Brazil; 10 ADHD Outpatient Program & Developmental Psychiatry Program, Hospital de Clínicas de Porto Alegre, Universidade Federal do Rio Grande do Sul, Porto Alegre, Brazil; 11 Child Mind Institute, New York, NY USA; 12 The Perelman School of Medicine, University of Pennsylvania, Philadelphia, PA USA; 13 Disciplina de Biologia Molecular, Departamento de Bioquímica, - Universidade Federal de São Paulo (UNIFESP), São Paulo, Brazil

**Keywords:** Genomics, Molecular neuroscience

## Abstract

Extracellular vesicles (EVs) are present in numerous peripheral bodily fluids and function in critical biological processes, including cell-to-cell communication. Most relevant to the present study, EVs contain microRNAs (miRNAs), and initial evidence from the field indicates that miRNAs detected in circulating EVs have been previously associated with mental health disorders. Here, we conducted an exploratory longitudinal and cross-sectional analysis of miRNA expression in serum EVs from adolescent participants. We analyzed data from a larger ongoing cohort study, evaluating 116 adolescent participants at two time points (wave 1 and wave 2) separated by three years. Two separate data analyses were employed: A cross-sectional analysis compared individuals diagnosed with Major Depressive Disorder (MDD), Anxiety disorders (ANX) and Attention deficit/Hyperactivity disorder (ADHD) with individuals without psychiatric diagnosis at each time point. A longitudinal analysis assessed changes in miRNA expression over time between four groups showing different diagnostic trajectories (persistent diagnosis, first incidence, remitted and typically developing/control). Total EVs were isolated, characterized by size distribution and membrane proteins, and miRNAs were isolated and sequenced. We then selected differentially expressed miRNAs for target prediction and pathway enrichment analysis. In the longitudinal analysis, we did not observe any statistically significant results. In the cross-sectional analysis: in the ADHD group, we observed an upregulation of miR-328-3p at wave 1 only; in the MDD group, we observed a downregulation of miR-4433b-5p, miR-584-5p, miR-625-3p, miR-432-5p and miR-409-3p at wave 2 only; and in the ANX group, we observed a downregulation of miR-432-5p, miR-151a-5p and miR-584-5p in ANX cases at wave 2 only. Our results identified previously observed and novel differentially expressed miRNAs and their relationship with three mental health disorders. These data are consistent with the notion that these miRNAs might regulate the expression of genes associated with these traits in genome-wide association studies. The findings support the promise of continued identification of miRNAs contained within peripheral EVs as biomarkers for mental health disorders.

## Introduction

Mental health disorders are among the 10 main causes of disability-adjusted life years (DALYs) globally. Depressive and anxiety disorders rank in fourth and sixth place as leading causes of disease burden in the global population between the ages of 10–24 years, and sixth and fifteenth in the population between the ages of 25-49 years [1].

These disorders are complex traits, in which both genetic and environmental risk factors contribute to the development of the trait. They are highly heritable [2, 3], polygenic and with pleiotropic genetic architecture [4, 5]. In this way, identifying molecular changes that are associated with mental health disorders provides additional information for understanding the underlying pathophysiology of these disorders to further validate diagnostic assessments and treatments. Furthermore, since the diagnosis of mental health disorders may involve different trajectories (e.g., individuals can convert or remit from diagnosis over time), longitudinal study designs are important to improve our understanding of changes in gene expression that may be involved in different stages of disorder course [6, 7]. A major limitation of understanding genetic mechanisms associated with mental health disorders is the inherent difficulty of accessing the brain of living individuals. Considering this, it is of great relevance to identify peripheral markers associated with risk for these conditions. To this end, extracellular vesicles (EVs), present in numerous bodily fluids, have been increasingly considered as important targets for molecular studies in psychiatry.

EVs are particles formed by a lipid bilayer and are secreted by all cell types and pathogens in all bodily fluids. They are produced by the majority of cells in the human body, including all cells in the nervous system (neurons, astrocytes, microglia, and oligodendrocytes) and can act in various manners, including in synaptic regulation and cell differentiation [8–11]. EVs function as important mediators of cell-to-cell communication, and have distinct subtypes, such as exosomes, microvesicles and apoptotic bodies, according to their origin, size, and cargo [12]. Exosomes are a subtype of small EVs (diameter 50-200 nm) that have their biogenesis from endosomes [13]. They have become targets of interest in research of neurological and psychiatric disorders because they are small enough to cross the blood-brain barrier [14]. For this reason, EVs have become targets of neurodegenerative disorder research because they can carry misfolded proteins associated with these conditions, disseminating these proteins across cells in the brain and propagating pathological processes [15]. Also for example, microRNAs (miRNAs) contained within EVs have been associated with the development of glioblastoma and Alzheimer’s disease, serving as potential biomarkers for the development of these conditions that can be detected in peripheral fluids [16, 17].

miRNAs are small transcripts (19-24 nucleotides) that target messenger RNAs and can regulate the expression of several genes at once [18–20]. They can be detected in biofluids such as serum, saliva and urine, both freely circulating or inside EVs, in which they are commonly enriched [21]. Research shows that miRNA carried by EVs can regulate gene expression in other cells, away from their cell of origin [22]. These molecules have been associated with mental disorders such as Major Depressive Disorder (MDD) [23–25], Anxiety disorders (ANX) [26, 27] and Attention deficit/hyperactivity disorder (ADHD) [28, 29].

Since changes in EV content can reflect pathologic processes, miRNAs contained in these vesicles may also serve as potential peripheral indicators of mental health disorders [30]. For example, a recent study demonstrated that higher levels of the miRNA miR-139-5p in EVs were associated with MDD and injecting EVs containing this miRNA from human MDD patients in mice resulted in subsequent depressive-like behaviors in the mice [31]. In another study, miRNAs in plasma EVs of MDD patients were associated with antidepressant treatment response [32].

Therefore, we conducted an exploratory evaluation of miRNA expression from serum EVs of adolescent participants and investigated associated pathways of the miRNA targets. Participants provided samples at two time points separated by three years. We organized the study to allow for two analysis approaches: cross-sectionally, to observe differences in expression associated with diagnostic status at each of the two-time points; and longitudinally, to observe changes in miRNA expression that occur along different trajectories of mental health disorder diagnosis. In the former analysis, we focused on MDD, ANX and ADHD, given that these are the most frequent mental health disorders in the studied cohort. Our aim for the cross-sectional comparisons was to document any differences in extracellular miRNA associated with mental health group status at each timepoint, regardless of future or past development of a mental health disorder.

## Methods

### Participants

We selected 116 participants from the Brazilian High-Risk Cohort Study for mental health disorders (BHRCS). Data were analyzed for the two data collection points (separated by three years) that followed participants’ baseline assessment as part of this larger study: Time point 1 is referred to as Wave 1 (w1) and Time point 2 is referred to as Wave 2 (w2) in this larger study. For diagnoses, participants were evaluated with the Development and Well-being Behavioral Assessment (DAWBA) and received a diagnosis according to the Diagnostic and Statistical Manual of Mental Disorders (DSM-IV).

The selection of individuals was based on blood serum sample availability for the analyses, and to represent four trajectory types of mental disorder diagnosis across the two timepoints in the longitudinal analysis, built according to the dichotomous diagnoses of psychiatric disorders according to DAWBA. We did not include the baseline assessment in this study due to a lack of individuals with baseline serum samples that could be paired with their w1 and w2 samples. To avoid selection bias, we balanced the selection of individuals regarding sex, age and location (considering that BHRCS recruitments were made in two different regions of Brazil), to have a study sample that reflected the whole cohort.

For the longitudinal analysis, the four trajectory groups were as follows: 1) Incidence group: individuals who transited to an incidence of any mental disorder diagnosis (N = 24, no diagnosis at w1, diagnosis at w2); 2) Remission group: remitted from any diagnosis (N = 26, diagnosis at w1, no diagnosis at w2); 3) Persistence group: persisted with any diagnosis (N = 39, any diagnosis at both time points); and 4) Control group: typically developing individuals who had not received a mental disorder diagnosis and did not make use of psychiatric medication (N = 27).

For the cross-sectional analyses, we considered diagnosis-no diagnosis status of MDD, ANX (Panic Disorder/Agoraphobia, Generalized Anxiety Disorder, Social Phobia and/or Specific Phobia) and ADHD participants at each time point. To elaborate, for the w1 analysis, the Incidence and Control groups were assigned to a “no diagnosis” condition, and the Remission and Persistence groups were assigned to a “diagnosis” condition. For the w2 analysis, the Control and Remission groups were assigned to a “no diagnosis” condition and the Incidence and Persistence groups were assigned to a “diagnosis” condition.

The BHRCS was approved by the Ethics Committee of Universidade Federal de São Paulo (CAAE: 74563817.7.2002.5505), Universidade Federal do Rio Grande do Sul (CAAE: 74563817.7.1001.5327) and Faculdade de Medicina da Universidade de São Paulo (IORG0004884), and all participants and their legal representatives provided written informed consent. This study was also approved by the Ethics Committee of Universidade Federal de São Paulo (CAAE: 28167420.6.0000.5505). Further cohort details are available elsewhere [33].

### Blood sampling

Blood samples were collected in BD Vacutainer Serum Separator Tubes (Becton Dickinson, NJ, USA) at both time points. After sample collection, tubes were centrifuged at 2000x *g* for 10 minutes at room temperature to separate the serum fraction. Serum samples were aliquoted and stored at -80°C until use.

### EV isolation and miRNA extraction

As recommended by the International Society of Extracellular Vesicles (ISEV), we used two methods for isolation of EVs [34]. EVs were isolated from 400 μL of serum using a differential centrifugation protocol followed by a precipitation kit (miRCURY Exosome Isolation Kit Serum/Plasma - QIAGEN, Hilden, Germany). Serum samples were centrifuged at 300x *g* for 10 minutes, 2,000x *g* for 30 minutes and 10,000x *g* for 45 minutes. All centrifugations were done at 4°C. In each step, the supernatant was carefully transferred to a new tube. After the last centrifugation, we carried out the precipitation kit protocol following manufacturer instructions. Afterwards, 200 μl of the isolated EVs were used for isolation of total RNA, including miRNA, using miRNeasy Advanced Serum/Plasma Kit (QIAGEN), and the remaining volume was stored at -20°C for EV characterization.

### EV Characterization

We performed two types of EV characterization to observe the characteristics of our total EV population in terms of a) size distribution and concentration and b) membrane markers.

- Measurements of size (nm) and concentration (particles/mL) of total EV samples were carried out by Nanoparticle Tracking Analysis (NTA) using NanoSight NS300 (Malvern Panalytical, UK). EVs were diluted in PBS before analysis (1:1000). Three 60 seconds recordings per sample were made and analyzed using the NTA software. Comparisons of size and concentration measures between groups were performed using RStudio.
- To detect the presence of EV membrane markers and check for the presence of neural-originated EVs, we used the antibody array Exo-check Exosome Antibody Array Neuro Standard (System Biosciences, Palo Alto, USA) for EV protein marker evaluation, previously quantifying total protein concentration of the isolated EV samples using Qubit protein assay kit (ThermoFisher Scientific, Waltham, USA) as indicated by the antibody array protocol, to determine quantity of EV sample to use for the array. We used 25 ng of protein as starting material and performed the antibody array as per the manufacturer’s protocol. At the end of the protocol, we used SuperSignal West Pico Plus Chemiluminescent Substrate (ThermoFisher Scientific) to reveal the membrane from the antibody array and acquired images on the Luminescent Image Alliance 4.7 (Uvitec, Cambridge, UK) imaging system.

### miRNA library preparation and sequencing

After isolation of RNA from EVs, libraries were prepared from 5 μl RNA with QIASEQ miRNA Library Kit (QIAGEN) according to the manufacturer’s protocol and diluting the 3’ adapters 1:10, 5’ adapters 1:3 and reverse transcription adapter 1:10, with 22 cycles for library amplification. Observations of library quality and size were performed with TapeStation High Sensitivity D1000 (Agilent, Santa Clara, USA). Libraries were sequenced on an Illumina NovaSeq 6000 sequencer (Illumina, San Diego, USA) with a 75 bp single end run.

### Sequencing data analysis

Sequencing quality was evaluated by FastQC and MultiQC (Babraham Bioinformatics). Adapter trimming was done through Atropos (version 1.1.31) [35]. Trimmed reads were aligned through the exceRpt pipeline for extracellular RNA-seq data [36]. Reads were preprocessed by aligning to UniVec contaminants and 45 S, 5 S and mitochondrial rRNA. Unmapped reads from this initial filtering were then aligned to the human genome (hg38), as well as miRNA (miRBase), tRNA (gtRNAdb), piRNA (piRNAbank), circRNA (circBase) and longRNA (gencode) databases. The read counts tables were imported to RStudio for analysis. Using DESeq2 (release 3.15) [37], we performed sample PCA analysis based on miRNA normalized read count data and plotted the results to observe possible clustering with sequencing batch, age, sex, and blood collection time – variables more likely to generate possible bias in the results. For differential expression analysis, we considered only miRNA with more than three reads in at least 75% of the samples for each comparison.

### In silico target prediction and pathway enrichment analysis

We used the miRWalk database (version 3, http://mirwalk.umm.uni-heidelberg.de/) [38] to map differentially expressed miRNA to their target gene transcripts. We considered for analysis targets with a binding probability > 0.95, reported in miRTarBase (experimentally validated targets) and that bind to either 3’ untranslated region (UTR), 5’UTR or coding DNA sequence (CDS) regions.

To observe if there is an overlap between mapped targets of differentially expressed miRNA and literature GWAS hits in each test, we extracted summary statistics table results from GWAS catalog (https://www.ebi.ac.uk/gwas/) for unipolar depression (207 GWAS Catalog “studies”), attention deficit/hyperactivity disorder (68 GWAS Catalog “studies”) and anxiety disorder (100 GWAS Catalog “studies”).

Pathway enrichment analysis was performed using Reactome v3.7 [39]. We considered pathways with an enrichment p-value < 0.05 as statistically significant results.

### Statistical Analysis

For the longitudinal analysis, we compared the repeated measures of all samples in a paired data model using the trajectory variable as an independent variable (between-subject effect) and the individual ID as a clustering variable (miRNA expression ~ Trajectory + Trajectory:ID + Trajectory:wave). In the cross-sectional analysis, we used a model with diagnostic status (MDD, ANX or ADHD) as the independent variable, sex and age as covariates, and compared diagnosis-no diagnosis status for each of the three disorders at each timepoint, separately (miRNA expression ~ age + sex + diagnostic status). We used Benjamini-Hochberg False Discovery Rate (FDR) for multiple comparisons correction (based on number of miRNAs analyzed) in the differential expression tests and considered as statistically significant the differentially expressed miRNAs with adjusted p-value < 0.1.

## Results

### Study sample

**Fig. 1:**
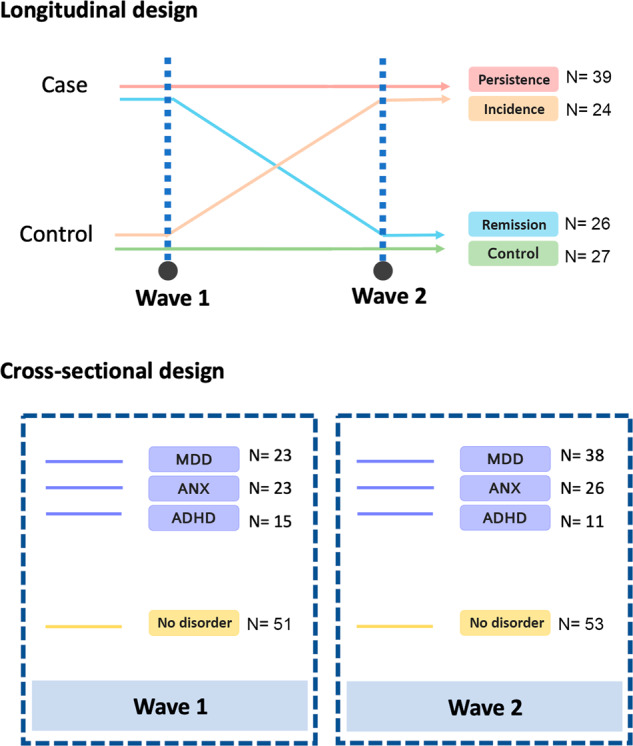
Schematic of the study design, illustrating the longitudinal and cross-sectional approaches. Longitudinal design: the dotted blue lines represent the two analyzed time points, Wave 1 and Wave 2. The colored horizontal linesrepresent the differences in case–control status between both time points,which was the criteria to define each trajectory group. Sample sizes for each group are also included. Cross-sectional design: the dotted blue lines represent each of the two time points that were analysed separately in this design. Inside each box, sample sizes for each considered disorder (MDD, ANX andADHD), as well as the no disorder group, are shown.

There were no statistically significant differences for sex or age (at w1 and w2) between trajectory-based groups. Table 1 summarizes the descriptive details of the studied sample and Fig. 1 summarizes the longitudinal and cross-sectional study designs. We included sample-by-sample information on sex, age, DAWBA diagnosis, use of psychiatric medication, and overlap between diagnoses in the Supplementary material (Supplementary Tables S1–S2 and Supplementary Fig. 1).

**Table 1:** Descriptive statistics of the study.

Variable	Control, *N* = 27	Incidence, *N* = 24	Persistence, *N* = 39	Remission, *N* = 26	*p* value^a^
**Biological Sex - N (%)**					0.6
Female	13 (48%)	12 (50%)	19 (49%)	9 (35%)	
Male	14 (52%)	12 (50%)	20 (51%)	17 (65%)	
**Age - Median (IQR)**					
W1	12.16 (11.80-13.38)	13.22 (11.34-14.28)	13.35 (11.81-14.92)	13.56 (11.72-15.33)	0.2
W2	16.42 (15.93-17.95)	17.70 (15.85-18.77)	18.00 (16.38-19.38)	17.89 (16.44-20.01)	0.2
**DAWBA - N (%)**	**W1/W2**	**W1/W2**	**W1/W2**	**W1/W2**	
ADHD	0/0	0/4 (17%)	6 (15%)/7 (18%)	9 (35%)/0	
ANX	0/0	0/11 (46%)	12 (31%)/15 (38%)	11 (46%)/0	
MDD	0/0	0/15 (62%)	15 (38%)/23 (59%)	8 (31%)/0	
Other*	0/0	0/1 (4%)	15 (38%)/7 (18%)	2 (7%)/0	
No mental disorder	27 (100%)/27 (100%)	24 (100%)/0	0/0	0/26 (100%)	

^a^Pearson’s Chi-squared test; Kruskal-Wallis rank sum test; * Presence of other mental disorders not described above (See Suppl. Table 1). W1: Wave 1; W2: Wave 2; IQR: Interquartile range SD: Standard deviation; DAWBA: Development and Well-Being Assessment; ADHD: Attention deficit hyperactivity disorder; ANX: Anxiety disorder; MDD: Major depressive disorder.

### EV Characterization

EV characterization through NTA indicated that they were within the range of small EV size (average mode of particle size (SD) = 194.8 nm (26.8 nm). There were no statistically significant differences between waves (w1 and w2 timepoints) and trajectories in mean size in nm (Wave: Paired T-test T = -2.1584, *p* value = 0.05386; Trajectory: ANOVA F = 0.2314, *p* = 0.8734) or concentration in particles/mL (Wave: Paired T-test T = 1.0064, *p* value = 0.3359; Trajectory: ANOVA F = 0.126, *p* = 0.944) (Supplementary Fig. 2).

Immunolabeling indicated the presence of general and brain-associated EV protein markers. We identified the presence of general membrane markers of small EVs CD81, CD9, TSG101 and ICAM1, but not CD63. In addition, we detected the presence of markers associated with brain EVs L1CAM, NCAM1, ENO2, MAPT, GRIA1, and PLP1. Finally, we did not observe the presence of CANX, a cytosolic protein, which would indicate presence of cellular components other than EVs in our samples (Supplementary Fig. 3).

### microRNA Analysis

Considering the 232 analyzed samples (116 paired samples), no batch effects were identified when comparing sex, timepoint, sequencing batch, trajectory, and period of blood collection (Supplementary Figs. 4–8).

**Fig. 2:**
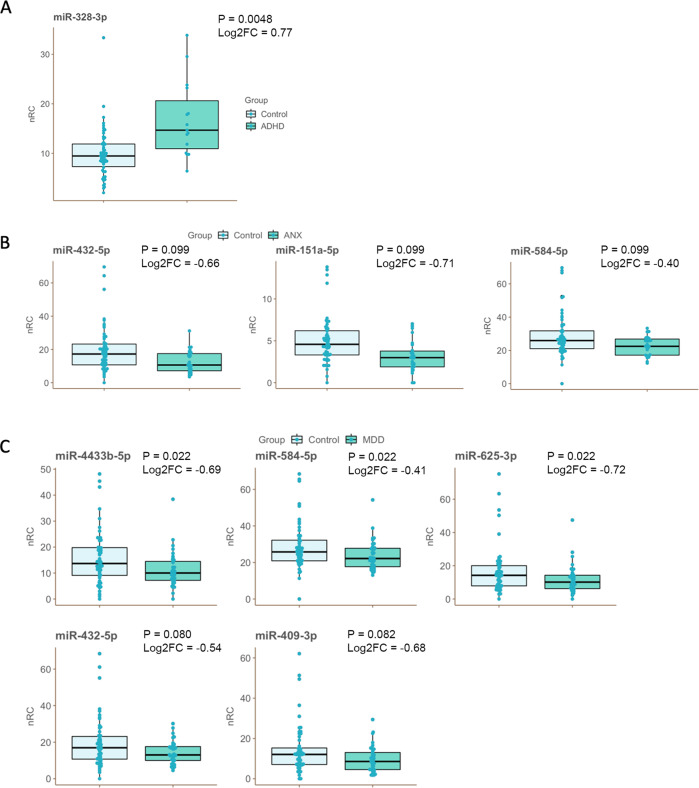
Normalized counts of differentially expressed miRNA. **A** miR-328-3p was upregulated in ADHD cases vs. no disorder controls in W1; **B** miR-432-5p, miR-151a-5p and miR-584-5p were downregulated in ANX cases vs. no disorder controls in W2; **C** miR-4433b-5p, miR-584-5p, miR-625-3p, miR-432-5p and miR-409-3p were downregulated in MDD cases vs. no disorder controls in W2. nRC = Normalized read counts. Significance values (FDR-adjusted *p* value) and effect size estimates (Log2 Fold Change - Log2FC) are shown beside each plot.

In the longitudinal comparisons, we did not observe statistically significant effects of wave or trajectory on miRNA expression (Supplementary Tables S3–S6). In the cross-sectional comparisons, in w1, considering a diagnosis of ADHD, we observed upregulation of miR-328-3p (*N* = 15) in comparison with individuals without a diagnosis at this time point (*N* = 51) (FDR-adjusted *p*-value = 0.0048, log2FC = 0.77) (Fig. 2A, Supplementary table S7). When considering a diagnosis of MDD or ANX, we did not observe statistically significant changes in miRNA expression at w1 (Supplementary tables S9 and S11).

At w2, when considering a diagnosis of ANX, we observed downregulation of miR-432-5p, miR-151a-5p and miR-584-5p (N_diagnosis_ = 26, N_no-diagnosis_ = 53, FDR-adjusted *p* value < 0.1) (Fig. 2B, Supplementary table S10). When considering a diagnosis of MDD, we observed downregulation of miR-4433b-5p, miR-625-3p, miR-584-5p, miR-432-5p and miR-409-3p (N_diagnosis_ = 38, N_no-diagnosis_ = 53, FDR-adjusted *p* value < 0.1) (Fig. 2C, Supplementary table S12). When considering a diagnosis of ADHD, we did not observe statistically significant changes at w2 (Supplementary table S8). Table 2 summarizes the significant results and Fig. 2 presents the boxplots of read counts for the differentially expressed miRNA. We performed additional analyses considering only the Control group (typically developing trajectory participants) as controls in the cross-sectional design (Supplementary Tables S20–S25).

**Table 2:** Summary statistics of the significant differentially expressed miRNA.

Comparison	microRNA	Test statistic	P-value	Adjusted P-value	log2 Fold Change
15 ADHD / 51 no disorder (w1)	miR-328-3p	4.197	2.70E-05	0.005 **	0.78
38 MDD / 53 no disorder (w2)	miR-4433b-5p	-3.662	0.00025	0.022 **	-0.69
	miR-584-5p	-3.546	0.00039	0.022 **	-0.41
	miR-625-3p	-3.568	0.00036	0.022 **	-0.72
	miR-432-5p	-3.112	0.00186	0.080 *	-0.54
	miR-409-3p	-3.778	0.00238	0.082 *	-0.69
26 ANX / 53 no disorder (w2)	miR-432-5p	-3.273	0.00106	0.099 *	-0.66
	miR-151a-5p	-3.208	0.00134	0.099 *	-0.72
	miR-584-5p	-3.142	0.00168	0.099 *	-0.4

* = adjusted *p* < 0.1; ** = adjusted *p* < 0.05.

### Target and pathway analysis

For miR-328-3p, which was upregulated in ADHD, 56 unique targets were found (Supplementary table S13). For the four miRNAs that were downregulated in ANX, 117 unique targets were mapped (Supplementary table S14). For the five miRNAs that were downregulated in MDD, 154 unique target transcripts were mapped (Supplementary table S15). Two targets were shared between two different miRNAs (*ELL2* and *STAMBP*). *STAMBP* was a common target for miRNA that were differentially expressed in MDD and ANX (miR-432-5p and miR-584-5p) (Supplementary table S16). Table 3 presents the 18 target genes of these miRNAs that overlap with GWAS hits for the respective traits they were associated with in this study.

**Table 3:** Targets of differentially expressed miRNA present in GWAS results.

miRNA	Target	Binding region^a^	GWAS Catalog trait	Study accession	Study	DOI
**miR-409-3p**	*CTNND1*	3’UTR	unipolar depression	GCST009600	Cross-Disorder Group of the Psychiatric Genomics Consortium, 2019	10.1016/j.cell.2019.11.020
**miR-432-5p**	*FMN1*	3’UTR, CDS	unipolar depression	GCST001850	GENDEP Investigators et al., 2013	10.1176/appi.ajp.2012.12020237
**miR-625-3p**	*IGSF9B*	3’UTR	unipolar depression	GCST005022	Zhou et al., 2017	10.1001/jamapsychiatry.2017.3275
**miR-432-5p**	*LRRC1*	3’UTR	unipolar depression	GCST009980	Coleman et al., 2020	10.1038/s41380-019-0546-6
**miR-432-5p**	*NBAS*	3’UTR, CDS	unipolar depression	GCST005904	Howard et al., 2018	10.1038/s41467-018-03819-3
**miR-432-5p**	*PCLO*	CDS	unipolar depression	GCST007342	Howard et al., 2019	10.1038/s41593-018-0326-7
**miR-4433b-5p**	*SLC12A5*	CDS	unipolar depression	GCST010416	Howard et al., 2019	10.1038/s41593-018-0326-7
**miR-432-5p**	*SETBP1*	CDS	unipolar depression	GCST007342	Coleman et al., 2019	10.1016/j.biopsych.2019.10.015
**miR-584-5p**	*TNFRSF21*	CDS	unipolar depression	GCST90096931	Thalamuthu et. al., 2022	10.1038/s41380-021-01379-5
**miR-4433b-5p**	*TRPM3*	3’UTR	unipolar depression	GCST009979	Thalamuthu et. al., 2022	10.1038/s41380-019-0546-6
**miR-432-5p**	*ZBTB20*	3’UTR	unipolar depression	GCST007102	Ho et al., 2018	10.1038/s41398-018-0246-z
**miR-432-5p**	*ZCCHC14*	CDS	unipolar depression	GCST001469	Ripke et al., 2013	10.1038/mp.2012.21
**miR-328-3p**	*ETV3*	3’UTR	attention deficit hyperactivity disorder	GCST000253	Anney et al., 2008	10.1002/ajmg.b.30871
**miR-328-3p**	*STT3A*	3’UTR	attention deficit hyperactivity disorder	GCST001877	Cross-Disorder Group of the Psychiatric Genomics Consortium, 2013	10.1016/S0140-6736(12)62129-1
**miR-328-3p**	*TMEM132B*	3’UTR	attention deficit hyperactivity disorder	GCST90061435	Karlsson Linnér et al., 2021	10.1038/s41593-021-00908-3
**miR-328-3p**	*TYW3*	3’UTR, CDS	attention deficit hyperactivity disorder	GCST90061435	Karlsson Linnér et al., 2021	10.1038/s41593-021-00908-3
**miR-432-5p**	*PCLO*	CDS	anxiety disorder	GCST009600	Cross-Disorder Group of the Psychiatric Genomics Consortium, 2019	10.1016/j.cell.2019.11.020
**miR-584-5p**	*TNFRSF21*	CDS	anxiety disorder	GCST000320	Otowa et al., 2019	10.1038/jhg.2008.17

^a^3’UTR: 3’ untranslated region; 5’UTR: 5’ untranslated region; CDS: Coding sequence.

Of 56 miR-328-3p targets, 41 were mapped to curated pathways; of these, we observed 19 statistically significant pathways (Supplementary table S17). Of these 19 pathways, we note the pathway “Defective SLC16A1 causes symptomatic deficiency in lactate transport (SDLT)” - *R-HSA-5619070* enriched by miR-328-3p’s target *BSG*, which represents 1 out of 9 genes in the pathway. We also note two pathways related to ion transport through voltage-gated channels: “Tandem of pore domain in a weak inwardly rectifying K + channels (TWIK)” - *R-HSA-1299308* and “Phase 3 - rapid repolarisation” *- R-HSA-5576890*, enriched by *KCNK6* (one out of four entities in the pathway) and *KCNH2* (1 out of 9 genes in the pathway), respectively.

Of 117 ANX-associated miRNA targets, 91 were mapped to curated pathways; of these, we observed 28 statistically significant pathways (Supplementary table S18). Since there were differentially expressed miRNA in common between ANX and MDD (miR-432-5p and miR-584-5p), there were pathways in common as well. Of these, we note the “C6 deamination of adenosine” - *R-HSA-75102*, and “Inhibition of voltage-gated Ca2+ channels via Gbeta/gamma subunits” - *R-HSA-997272*, enriched by *KCNJ6* and *GNG12*.

Of 154 MDD-associated miRNA targets, 123 were mapped to curated pathways (Supplementary table S19). Among the 23 significant pathways, we note those related to GABA receptor activation: “GABA receptor activation” - *R-HSA-977443*, “GABA B receptor activation” - *R-HSA-977444*, and “Inhibition of voltage-gated Ca2+ channels via Gbeta/gamma subunits” - *R-HSA-997272*, enriched by *KCNJ6, GNG12* (targets of miR-584-5p) and *GNAL* (target of miR-409-3p), which represent three out of 68 genes, three out of 50 and two out of 31 genes, respectively.

## Discussion

In this study, we analyzed miRNAs of serum extracellular vesicles isolated from adolescent participants with mental health disorders. We were able to characterize EV membrane proteins, identifying the presence of neural markers besides general EV markers in our samples. In the longitudinal analysis, we did not observe associations between trajectories of mental health disorders and miRNA expression. In the cross-sectional analysis, we observed one differentially expressed miRNA in ADHD at w1, five in MDD at w2, and three in ANX at w2. Here we discuss the details and implications of these findings.

### Cross-sectional analysis

In participants with ADHD, miR-328-3p was upregulated; among the enriched pathways of its targets, we observe that *BSG* (Basigin) is expressed in the nervous system and regulates a pathway involved in the disruption of monocarboxylate transporter 1 (SLC16A1) that leads to lactate transport deficiency [40]. SLC16A1 is enriched in neuroglia cells, and its disruption may lead to neuron damage through a decrease in axon support from oligodendrocytes and astrocytes [41]. One of the suggested pathways disrupted in the neurobiology of ADHD is the lactate transport from astrocytes to neurons: lactate is an energy source for neurons, and an impairment in lactate supply reduces glutamate neurotransmission [42]. We speculate that a disruption in this pathway mediated by an upregulated miR-328-3p could be a mechanism through which this miRNA acts in ADHD.

In participants with MDD and ANX, miR-432-5p was downregulated; miR-432-5p regulates the expression of *ADAR1*, which is suggested to be involved in processes associated with MDD [43]. *ADAR1* is highly expressed in brain, and it is involved in pre-mRNA editing through deamination of adenosine. This mechanism occurs in mRNAs that codify ion channels such as the glutamate and serotonin (5HT2C) receptors, altering their ion conductance and consequently, neurotransmission [44]. A downregulation of miR-432-5p, as observed in our study, may affect the regulation of glutamate and serotonin pathways and lead to alterations involved in these disorders. This miRNA has also been associated with schizophrenia, with lower expression in patients in comparison with controls [45]. Both miR-432-5p and miR-328-3p have been previously observed to be upregulated in a study of autism, further suggesting a role of these miRNA in pathways related to neurodevelopment [46].

In participants with MDD, miR-409-3p was downregulated. A previous study demonstrated that this miRNA was downregulated in brain samples of individuals with MDD who had committed suicide [47]. It has been suggested that miR-409-3p has a role in the development of cortical neurons, specifically, neuron subtype specification [48]. Indeed, this miRNA is believed to play a critical role in the regulation of genes involved in neural integrity and synaptic plasticity, as its increased expression has been shown to be associated with improved cognitive function in humans and mice [49]. In addition, one of miR-409-3p’s target transcripts, *ATXN3*, is involved in processes that lead to apoptosis in neurons, and an overexpression of miR-409-3p decreased apoptosis in a cell model [50]. Thus, this miRNA may play a role in neurogenesis and promote protective mechanisms in neurons, and its downregulation, as observed in our study, could be related to mechanisms that are dysregulated in the brain of individuals with MDD.

This more global role for miR-409-3p fits with one additional finding in the present study: one of miR-409-3p’s targets, a risk loci in Delta-catenin 1 gene (*CTNND1*), was identified in a GWAS as being shared between multiple mental health disorders, and it was one of the 23 loci with the highest number of cross-disorder associations (MDD, ADHD, Autism spectrum disorder, Obsessive-compulsive disorder and Schizophrenia) [51]. CTNND1 is a protein involved in cell-to-cell junctions and signal transduction, and has a role in the establishment of the synaptic junction itself [52, 53]. Interestingly, in our study, miR-409-3p was associated with MDD and was also one of the top 5 differentially expressed miRNAs for ANX and ADHD (although not statistically significant – Supplementary tables S2 and S3). This statistical trend may suggest a role for miR-409-3p in the overall risk for mental health disorders that is shared between different disorders, consistent with the observed *CTNND1* GWAS hit overlap.

We note that the targets of miR-584-5p and miR-409-3p, two miRNAs that were decreased in ANX and MDD in our study, regulate pathways involved with GABA receptor activation. Multiple studies have observed reduced functionality in GABAergic neurotransmission in MDD patients [54]. Our data offer preliminary evidence that dysregulations in gene expression in the GABA pathway driven by these miRNAs may be part of the alterations that lead to deficits in the GABAergic system in MDD and ANX.

Variants in *TNFRSF21*, one of the targets of miR-584-5p, which was decreased in MDD and ANX in our study, were previously associated with MDD (rs188552424) [55] and a statistical trend toward an association with panic disorder (rs2103868) in GWAS [56]. This gene is expressed in brain and is a negative regulator of oligodendrocyte maturation [57]. A downregulation of miR-584-5p, as observed in our results, could promote a higher expression of *TNFRSF21* that would induce oligodendrocyte death and axon degeneration.

It is worth noting that not all of our results were consistent with previous findings: miR-4433b-5p was previously observed to be upregulated in MDD patients in comparison with controls [58], while our results pointed to decreased expression of this miRNA. Although the direction of effect in this study contrasts our findings, both results point to a role of this miRNA in regulating genes and pathways involved with MDD.

We observed no previous associations of miR-151a-5p and mental health disorders in the literature. This miRNA was downregulated in ANX in our study, and has the potential to be a molecular indicator for ANX that should be validated and further explored in further studies.

### Longitudinal analysis

We observed no statistically significant results in the longitudinal comparison. This may be due to a lack of statistical power to detect differences in the longitudinal design. It is important to note that there is a gap in the literature for longitudinal studies that analyze these conditions in youth while assessing EV-miRNA expression, and the results presented here may be used in future meta-analysis studies or as priors for Bayesian analyses.

### Limitations

We followed methodology and data reporting recommendations from the International Society for Extracellular Vesicles [34], allowing for replication by other research groups. However, this study has limitations that must be considered. First, the results found in serum EVs involve multiple tissue EVs and the correlation between the amount and types of miRNAs found in the nervous system and those found in circulating EVs is not yet clearly established in the field. Therefore, the current study does not address whether the differentially expressed miRNAs observed here reflect a state similar to that of neural cells. That said, the present results provide the field with initial testable results implicating these miRNAs with specific mental health disorders. Second, it is not yet established exactly which factors influence extracellular vesicle miRNA expression; we took into consideration age and sex as covariates, but other covariates might play an important role. It is relevant to note that fasting and blood collection times were not uniform in the BHRCS and this may have had an impact on the extracellular vesicle profile. Although we performed PCA analysis to check for the possibility of considerable clustering in regards to blood collection time, we cannot rule out the possibility that these variables did not impact the data. Third, we note the inherent difficulty associated with comorbidity when selecting a study sample for a study of mental health disorders. Overlap of diagnoses with other psychiatric conditions in an individual is a common occurrence in psychiatry [59], and therefore, we cannot rule out the potential impact of comorbidity on the results. We have included summary statistics of the differential expression analyses, and further sample information on comorbidity and treatment, in the supplementary material for the consideration of future studies and meta-analyses.

## Conclusion

Here, we presented a miRNA profile from serum EVs of adolescent participants with mental health disorders. The results identified numerous miRNAs, some consistent with previous studies and others novel to this study, that were associated with the presence of MDD, ANX and ADHD. Our data are largely consistent with previous findings in the literature that these miRNAs regulate genes involved in biological processes and regulatory mechanisms that have been previously implicated in these disorders. This work is relevant for identifying peripheral miRNAs associated with the establishment of mental health disorders, focusing on individuals during adolescence, a critical period for disorder development [60], and these data provide research targets for future studies that can assess their potential as biomarkers for mental health disorders and determine whether these results can be replicated in brain tissue or neuron-derived EVs.

## Supplementary information

Supplementary Figures

Supplementary Tables S1-S25

## Data Availability

Statistical analyses were conducted using R version 4.1.2: we used the R packages DESeq2 for all differential expression analyses and principal component analyses, ggplot2 to plot results, gtsummary to make the descriptive statistics table, VennDiagram to plot the disorder overlap Venn diagram, and dplyr for general data tidying and table manipulation. Code used to generate the results is available at https://github.com/mauerjh/mirna-expression.
