# Supplementary material for: Alterations in microRNA of extracellular vesicles associated with major depression, attention-deficit/hyperactivity and anxiety disorders in adolescents
Source: Transl Psychiatry. 2023 Feb 6;13:47. doi: 10.1038/s41398-023-02326-4 (PMC9902559; doi:10.1038/s41398-023-02326-4)
Supplement: Supplementary file 1 — Supplementary Figures [file 41398_2023_2326_MOESM1_ESM.docx]

**Supplementary Figures for Honorato-Mauer et al.**

**
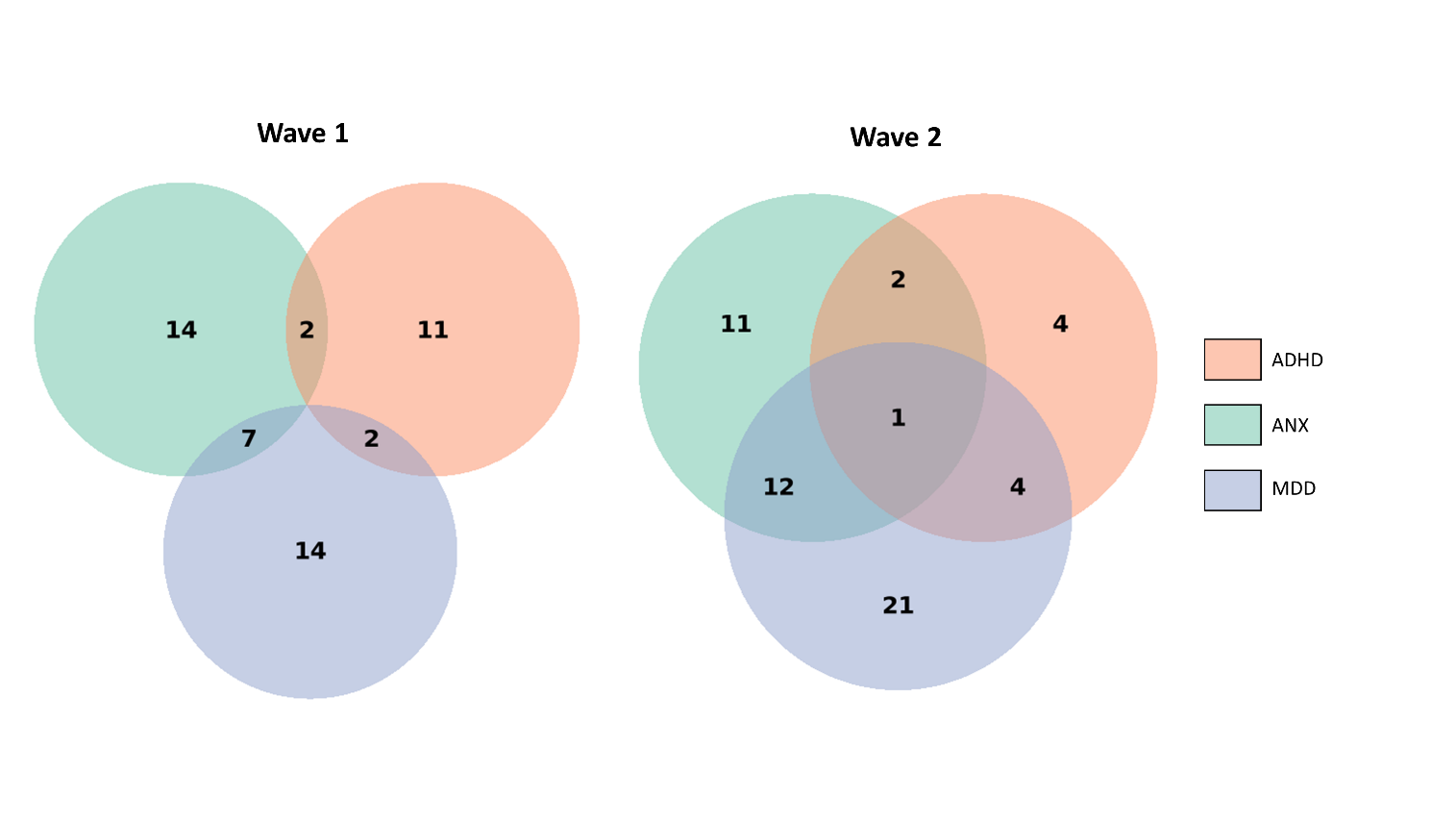
**

**Supplementary figure 1.** Number of participants of the study that were diagnosed with ADHD, ANX and/or MDD, separated by time point (Wave 1 - w1 and Wave 2 – w2).


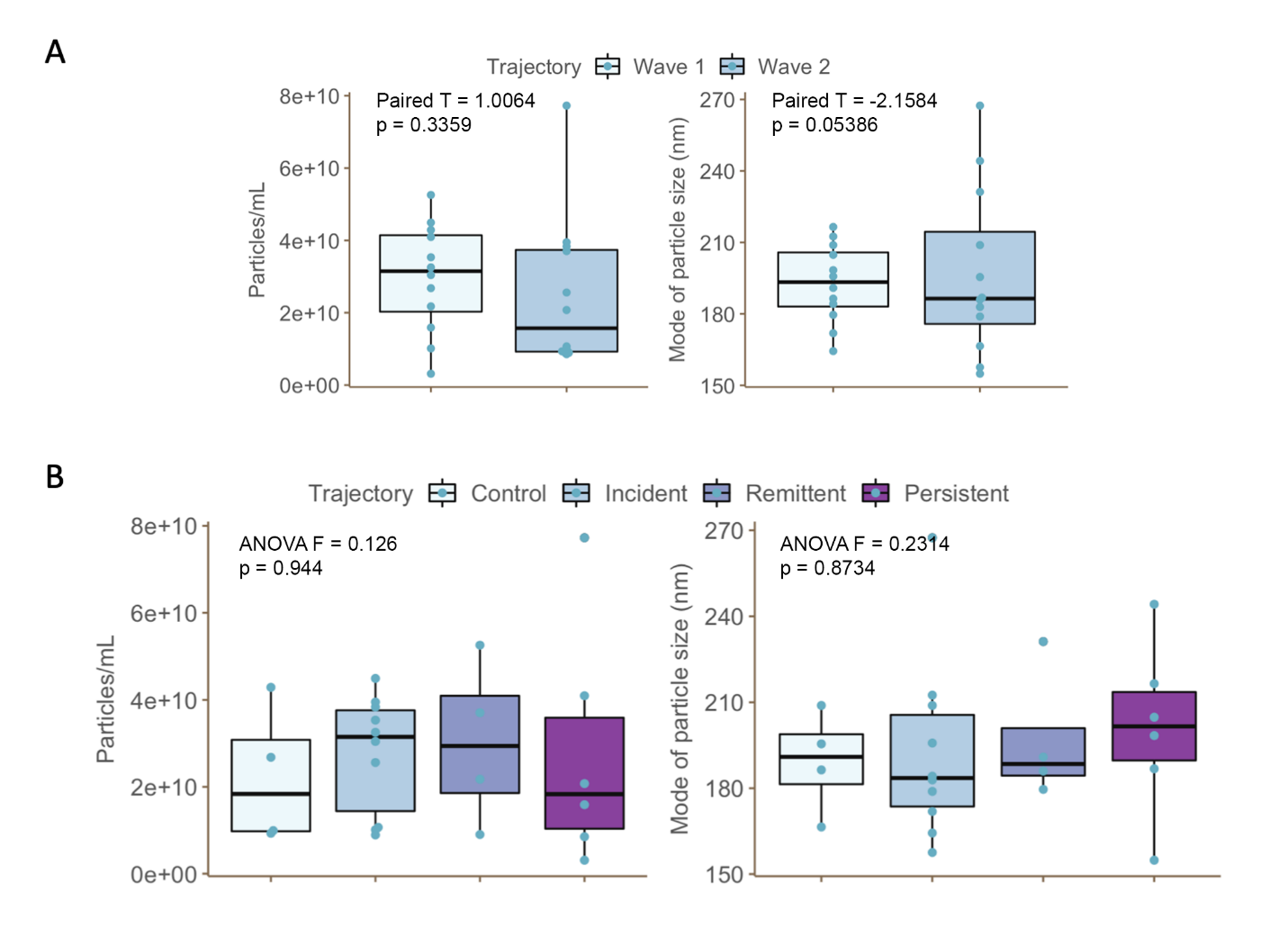


**Supplementary Figure 2**: Boxplots of concentration and mode of particle size between A) Waves and B) Trajectories, and values of comparisons of mean size in nm (Wave: Paired T-test T = -2.1584, p-value = 0.05386; Trajectory: ANOVA F = 0.2314, p = 0.8734), and concentration in particles/mL (Wave: Paired T-test T = 1.0064, p-value = 0.3359; Trajectory: ANOVA F = 0.126, p = 0.944).


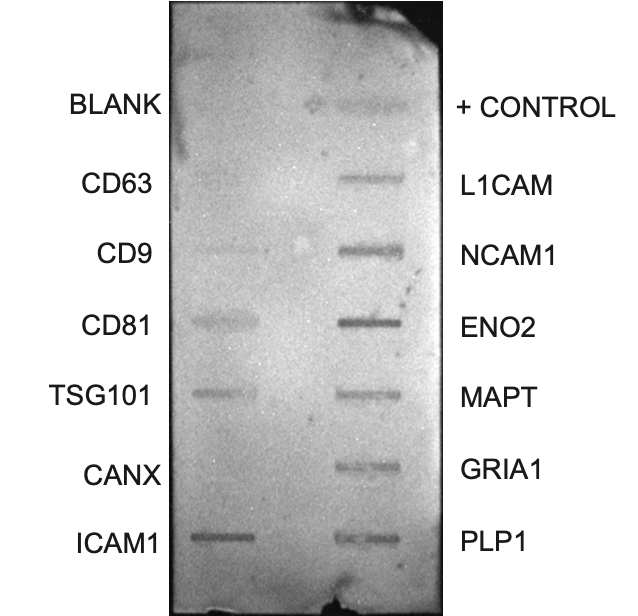


**Supplementary Figure 3**: Revealed image of protein antibody array membrane indicating presence of general (left) and brain-associated (right) EV membrane markers present in our samples.


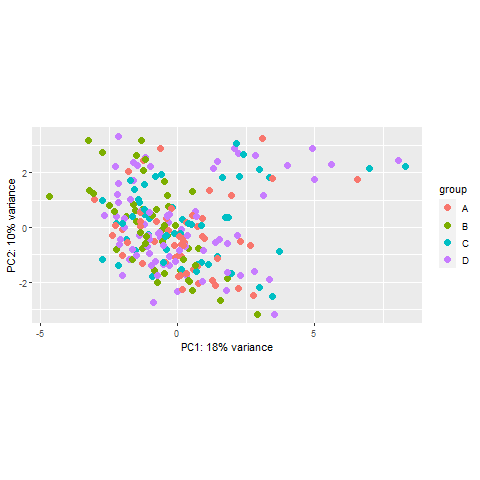


**Supplementary Figure 4.** Principal Component Analysis plot (PC1 x PC2). Dots are labeled according to trajectory group. A = Control, B = Incident, C = Remitting, D = Persistent.


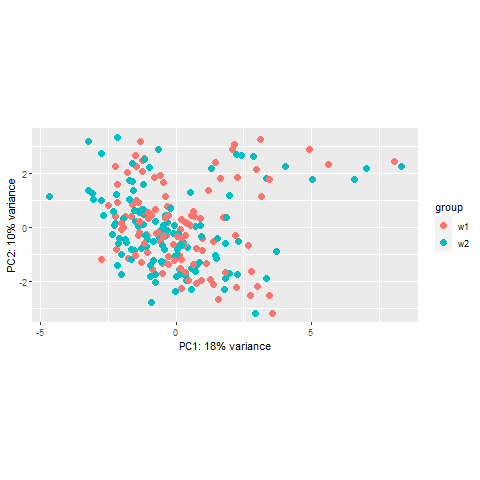


**Supplementary Figure 5.** Principal Component Analysis plot (PC1 x PC2). Dots are labeled according to time point. w1 = Wave 1, w2 = Wave 2.


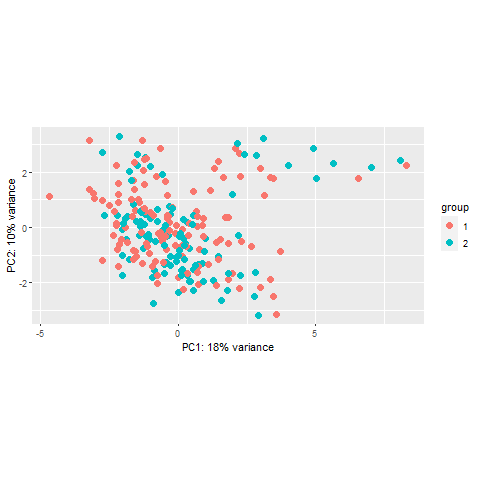


**Supplementary Figure 6.** Principal Component Analysis plot (PC1 x PC2). Dots are labeled according to sex. 1 = male, 2 = female.


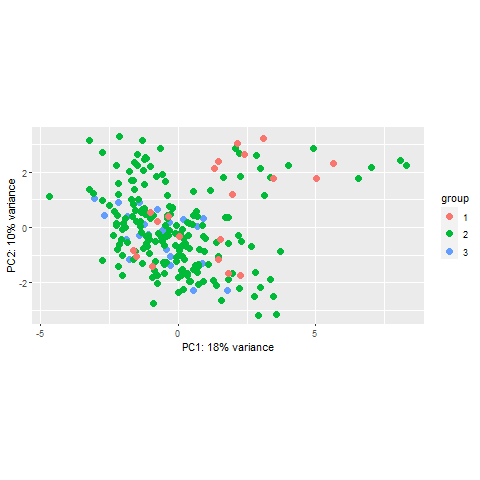


**Supplementary Figure 7.** Principal Component Analysis plot (PC1 x PC2). Dots are labeled according to 1st, 2nd or 3rd miRNA sequencing batch.


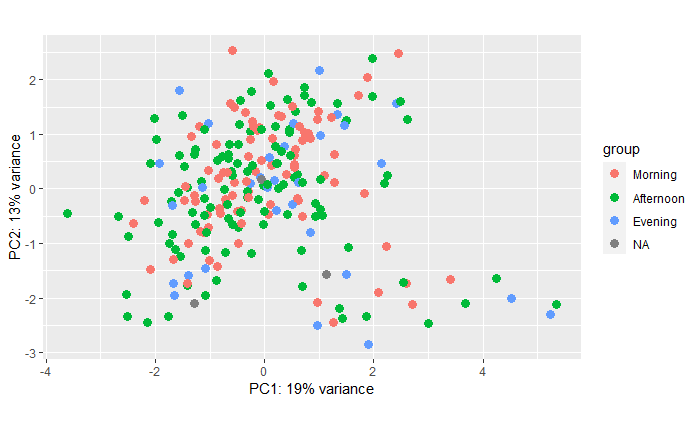


**Supplementary Figure 8.** Principal Component Analysis plot (PC1 x PC2). Dots are labeled according to period in which the participants had their blood samples collected (morning, afternoon, Evening). NA = data not available.
